# Current Findings Regarding Natural Components With Potential Anti-2019-nCoV Activity

**DOI:** 10.3389/fcell.2020.00589

**Published:** 2020-07-03

**Authors:** Jin Zhou, Jie Huang

**Affiliations:** ^1^Shenzhen International Graduate School, Tsinghua University, Shenzhen, China; ^2^Network of Aquaculture Centres in Asia-Pacific, Bangkok, Thailand; ^3^Laboratory for Marine Fisheries Science and Food Production Processes, Qingdao National Laboratory for Marine Science and Technology, Yellow Sea Fisheries Research Institute, Qingdao, China

**Keywords:** 2019-nCoV, structural feature, natural products, functional mechanisms, therapeutic strategies

## Abstract

COVID-19, a novel coronavirus pneumonia (named by the World Health Organization, WHO), has spread widely since the end of 2019. Research on synthetic drugs and vaccines has become a focus of attention in China and other countries, as such approaches are regarded as key tools for disease prevention and control; however, the development of these therapeutics will take months, or even years. Under such circumstances, development of coronavirus specific therapeutics is urgent. For this specific indication, the rapid performance of natural products, such as plant compounds, herbal extracts, and traditional Chinese medicine, could contribute as alternative measures. Recent investigations have provided evidence that these natural products are potential candidates for development as therapeutic agents against the virus that causes COVID-19, 2019-nCoV. Targeting the structural proteins or cellular receptors of 2019-nCoV, including coronavirus chymotrypsin-like (3CL^pro^ or M^pro^), helicase (nsP13), S protein, and human angiotensin converting enzyme 2 (ACE2), holds promise for preventing infection. In this review, we summarize some representative natural products and their active components that have potential anti-2019-nCoV effects. We focus on the basic structural elements of 2019-nCoV, its main mechanisms of action, and the feasibility and potential of products to inhibit the novel coronavirus. In addition, the relative advantages, additional functions, and precautions that should be used with typical natural products are also discussed. The aim is to make the case that natural products could be a valuable pool for the development of active compounds for treating 2019-nCoV infection, which may contribute to mitigation of the spread of the pandemic.

## Introduction

A recent outbreak of coronavirus named “2019 novel coronavirus (2019-nCoV)” has occurred in Wuhan. This novel β-coronavirus (Phan, 2020) was identified on 7 January 2020, its taxonomy is a strain of the species of Severe acute respiratory syndrome-related coronavirus named as SARS-CoV-2 (Gorbalenya et al., 2020). The newest data show that 2019-nCoV originates from bats (Cui et al., 2019; York, 2020; Zhou et al., 2020a). The current situation is driving urgent public health actions, as well as international engagement of scientists (Du Toit, 2020). Ongoing investigations are focusing on understanding the epidemiology, molecular biological characteristics, evolutionary history, and methods to combat transmission (Guan et al., 2020); however, the most urgent need is to understand the mechanisms of transmission and clinical manifestations, develop diagnostic technology, and implement global risk assessment and therapeutic strategies (Kruse, 2020; Wrapp et al., 2020).

By 14 June 2020, this highly contagious sickness had caused over 7,690,708 confirmed cases and killed 427,630 people in 213 countries, including China, Iran, South Korea, Japan, Italy, Spain, France, UK, the United States, Canada, Brazil, Egypt, Australia, and other countries in Americas, Eastern Mediterranean, Europe, South-East Asia, Western Pacific, and Africa (WHO, 2020). Unlike SARS and MERS, infection with 2019-nCoV has a relatively long incubation period (Guan et al., 2020). Treatment of these coronaviruses in outbreak settings has focused on general quarantine and physical isolation methods or antiviral treatment. For the former, the newest modeling results indicate that quarantine (for example, travel restrictions) only modestly influences the epidemic trajectory, unless paired with public health interventions and behavioral changes that achieve a considerable reduction in disease transmissibility (Chinazzi et al., 2020). For the latter, at present, clinical and laboratory studies have found that there are some chemicals may have a potential effect against 2019-nCoV infection; for example, lopinavir/ritonavir (KALETRA®), remdesivir, abietol, and chloroquine, among others (Li and Clercq, 2020; Lu, 2020). These antiviral drugs are prescription drugs, and their prescription requires medical diagnosis of a suspected or confirmed cases after symptoms appear. Further, the availability and price of these chemicals fundamentally limit their use. In addition, although several international organization working on the development of vaccines and antiviral agents to prevent and treat 2019-nCoV, effective medicines are not yet available, and development of these treatments may require months or even years. Hence, based on the current situation, we deem that a more immediate treatment, or alternative strategies, should be used where possible.

Natural products (such as plant extracts, traditional Chinese medicine, and herbs) present a potentially valuable resource against this virus. In fact, since the outbreak of SARS, many anti-coronavirus agents have been found among natural compounds, including some plant compounds and traditional Chinese herbal medicines (Wu et al., 2004; Li et al., 2005b; Park et al., 2017). The effectiveness of natural products for treatment aiming to control pneumonia disease has been demonstrated during the 2019-nCoV treatment period in recent days (Zhang et al., 2020). Use of herbal medicines has been encouraged for shelter hospitals in Wuhan to fight this new viral pneumonia. Some herbal medicines have very good efficacy in combination with western medicine, and a proportion have entered the clinical trial stage following *in vitro* experiments (Xia et al., 2020). Meanwhile, from the viral molecular structure, the coronavirus encodes at least a dozen proteins, including papain-like protease (PL^pro^), 3C-like protease (3CL^pro^), and spike protein (S protein). These functional units are essential for viral entry and replication, and their characteristics make them attractive targets for drug development. Previously, various active molecules, including those from natural compounds, have been identified by *in silico* and biological screening and demonstrated to directly blocking these functional proteins in SARS or MERS coronaviruses (Wen et al., 2007; Shen et al., 2019). The genetic sequence of 2019-nCoV has high homology with SARS-CoV and MERS-CoV (Chen et al., 2020). Hence, previously reported against SARS-CoV or MERS-CoV natural compounds probably become a useful reference to assist identification of anti-2019-nCoV natural products that can treat the viral pneumonia.

As efficient strategies against coronavirus, compared with chemical drugs, natural medicines (plant extracts, herbs, medicinal foods, marine peptides, and active small-molecule compounds) are readily available and highly cost-effective. Facing the severity of the 2019-nCoV outbreak, we mainly discussion the potential to repurpose existing natural antiviral products for treating infections caused by the agents of SARS, MERS, and COVID-19. Simultaneously, it should be noted that the application of herbal treatments is mainly based on the catalog of classical literature on herbs and the patient's symptoms. There is usually not enough information to predict whether these herbs can directly target the cause of viral disease. Therefore, based on the above analysis, in this article we review current plant natural products and their antiviral mechanisms of action and discuss their use from a viral pathology perspective. We hope this will compile current information for people to consider self-management with natural components after a high-risk exposure to 2019-nCoV without available hospital treatment. Furthermore, from a system perspective, we wish to offer new alternative strategies for public health workers, infrastructure managers, and decision makers to use natural products as potential pool of medicines to control 2019-nCoV (Ganasegeran and Abdulrahman, 2020; Wang et al., 2020; Zhu et al., 2020).

## The Basic Structure Of 2019-nCOV Indicates the Possibility for Application of Selected Herbal Medicines

The 2019-nCoV genome is 29870 bp (GenBank MN908947) and encodes five typical open reading frames, including ORF1ab polyprotein [7096 amino acids (aa)], spike glycoprotein (1273 aa), envelope protein (75 aa), membrane protein (222 aa), and nucleocapsid protein (419 aa) (Chen et al., 2020). Four kinds of non-structural proteins are the key to viral replication and CoVs infection. Homotrimers of S proteins comprise the spikes on the surface of virus particles, which are keys for viral attachment to host receptors (Ujike et al., 2016). There are 3 transmembrane domains in M protein. These domains can shapes the virions, promotes membrane bending, and binding with nucleocapsid (Neuman et al., 2011). The E protein functions in virus assembly and release, and is required for pathogenesis (Nieto-Torres et al., 2014). The N protein contains 2 functional domains, both of them can bind the virus RNA genome by different pathways. In addition, a structural protein (spike glycoprotein) is also present in this virus. These four non-structural proteins are the key enzymes in the life cycle of virus, and the spike glycoprotein is necessary for interactions of virus–cellular receptor in the process of viral entry (Zumla et al., 2016). These five proteins are therefore recognized as attractive targets for the development of antiviral agents against SARS and MERS (Zumla et al., 2016).

From its sequence, catalytic sites in 2019-nCoV enzymes appear to be highly conserved and share highly sequence similarity with the reported SARS-CoV and MERS-CoV enzymes (Morse et al., 2020). The main drug-binding pockets in structural viral proteins are also probably conserved across 2019-nCoV, SARS-CoV, and MERS-CoV (Morse et al., 2020). Additionally, structural analysis suggests that the 2019-nCoV cellular receptor in humans, angiotensin converting enzyme 2 (ACE2)/B0AT1 complex can bind two S-protein at the same time, providing important inspiration for recognition and infection with coronaviruses of the subgenus *Sarbecovirus* (genus *Betacoronavirus*) (Zhou et al., 2020b). Consequently, it is reasonable to consider repurposing existing MERS-CoV and SARS-CoV natural inhibitors for use against 2019-nCoV (Li and Clercq, 2020; Wu A. et al., 2020). At present, several herbal or food medicines of plant origin have been identified as effective in clinical treatment to inhibit infection with 2019-nCoV in clinical studies, or have shown promising progress in laboratory studies of viral infection (Ling, 2020; Zhang et al., 2020). Once approved by the relevant authorities, these drugs may be used as emergency prevention and clinical treatment drugs against 2019-nCoV. Therefore, use of these herbal medicines or food ingredients for self-medication/dietary management can be considered.

## Natural Components and Mechanisms of Action Against 2019-nCOV

**Figure 1:**
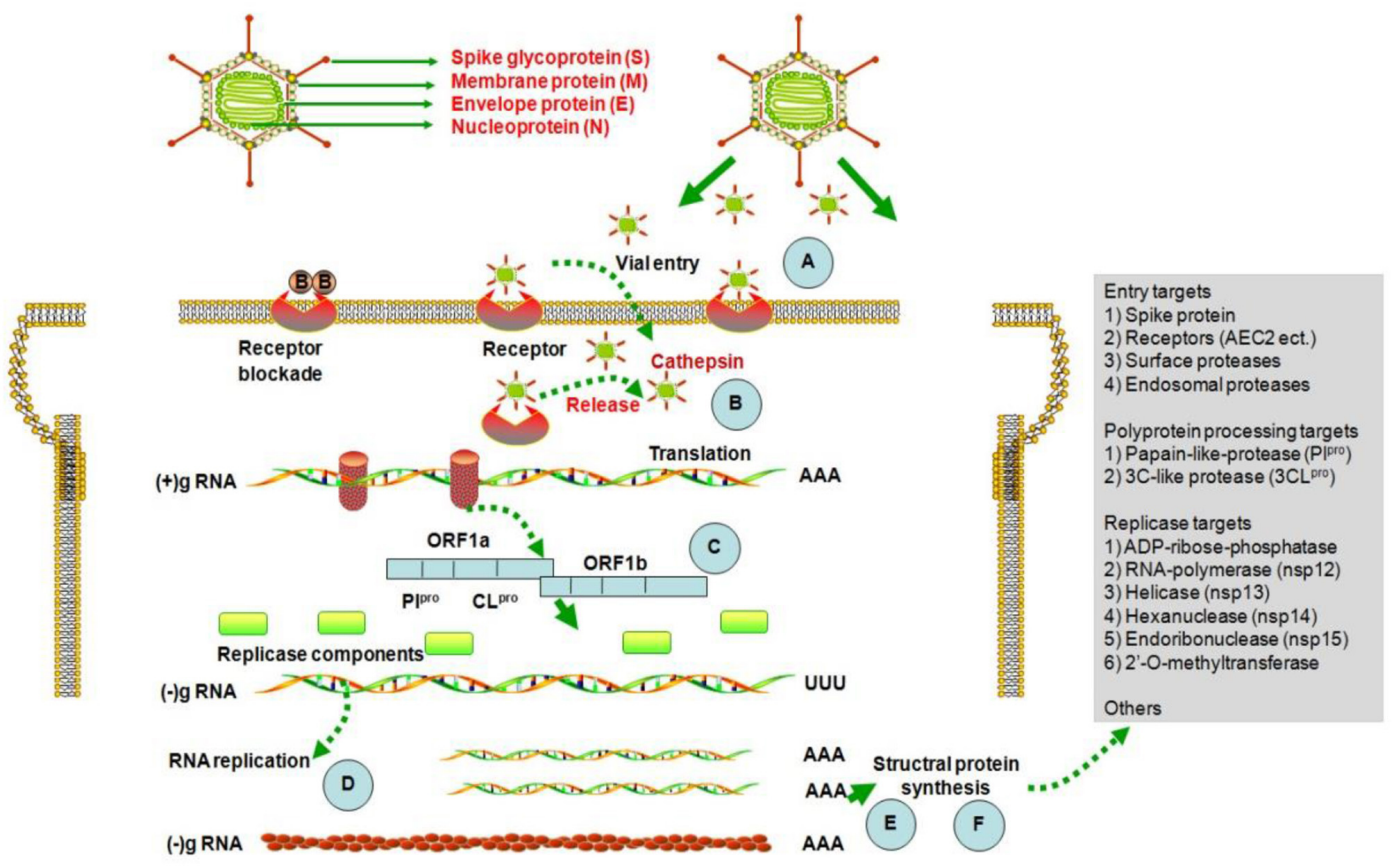
Structure of coronavirus and potential mechanisms of activity of natural products against them. 2019-nCoV utilizes host cellular components to achieve various physiological processes, including viral entry, genome replication, and the assembly and budding of virions. Therefore, interrupting any stages of the viral life cycle (A–F) is a potential therapeutic target for developing antiviral therapies (Pillaiyar et al., 2020).

After the outbreak of SARS in 2003, researchers screened various natural active components for inhibition of the SARS coronavirus, and the resulting data can be used for reference in efforts to prevent and control 2019-nCoV (http://apps.who.int/medicinedocs/en/d/Js6170e). The main screening strategies were based on tests of coronavirus infection inhibition activity *in vitro*, including assessment of cytopathogenic effect (CPE) or plaque forming units (PFU), and inhibition of the activity of viral enzymes, including the 3CL^pro^ protein, nsP13. Computer technologies were also used to identify natural components with potential to bind to the 2019-nCoV cellular receptor, ACE2 (Zhang et al., 2020). The possible mechanisms of activity of natural components against 2019-nCoV are presented in Figure 1.

### Natural Components With *in vitro* Coronavirus Infection Inhibition Activity

Multiple natural components have been tested for CPE inhibiting activity (Table 1). Two components, lycorine, and *Allium porrum* agglutinin (APA), showed very strong average inhibition activities, The former with 50% effective concentrations (EC_50_) was 15.7 ± 1.2 nM (0.00451 ± 0.00034 μg/ml) (Li et al., 2005b). The later showed EC_50_ values at 0.45 ± 0.08 μg/ml, and a significant correlation (*r* = 0.70) was found between the EC_50_ values of this plant lectins effective against the SARS-CoV (Keyaerts et al., 2007). Notably, the 50% cytostatic concentrations (CC_50_) of most components were >100, indicating low toxicity. The selective index (SI) values of lycorine and APA, calculated as the ratio of CC_50_ and EC_50_, were >200, indicating a very large potential dose selection for clinic trials (Keyaerts et al., 2007). However, plant agglutinins are proteins, which are difficult to be absorbed by oral administration. Lycorine, reserpine, and escin (Aescin), have important roles in the prevention and treatment of new respiratory infectious diseases, such as SARS and MERS (Wu, 2004; Li et al., 2005b; Shen et al., 2019). The natural product, silvestrol, is also an effective and biosafety inhibitor of cap-dependent viral mRNA translation in CoV-infected model cells (i.e., human embryonic lung fibroblast cells), and was highly effective against both infections, with EC_50_ values of 1.3 and 3 nM, respectively. Mechanistically, silvestrol strongly inhibits the formation of viral replication/transcription complexes by down-regulation the expression of CoV structural and non-structural proteins (nsp8) (Muller et al., 2018). Recently, Shen et al. (2019) identified seven compounds (lycorine, emetine, monensin sodium, mycophenolate mofetil, mycophenolic acid, phenazopyridine, and pyrviniumpamoate) from high throughput screening as wide-spectrum inhibitors, according to their strong inhibition of replication by four CoVs *in vitro* at low dose. These seven wide-spectrum inhibitors suppressed all CoVs' replication in a dose-dependent fashion and with low EC_50_ values; however, before they can be applied clinically, the efficacy and safety of these components for treatment of 2019-nCoV requires further confirmation in clinical trials.

**Table 1:** Natural components that potentially inhibit SARS-CoV CPE.

**Components**	**Categories**	**EC_**50**_ (μg/ml)**	**CC_**50**_ (μg/ml)**	**SI**	**References**
Lycorine	Alkaloids	(4.51 ± 0.34) × 10^−3^	4.3077 ± 0.2621	>900	Li et al., 2005b
APA	Agglutinins	0.45 ± 0.08	>100	>222.2	Keyaerts et al., 2007
UDA	Agglutinins	1.3 ± 0.1	>100	>78.8	Keyaerts et al., 2007
Morniga M II	Agglutinins	1.6 ± 0.5	>100	>62.5	Keyaerts et al., 2007
Nictaba	Agglutinins	1.7 ± 0.3	>100	>58.8	Keyaerts et al., 2007
EHA	Agglutinins	1.8 ± 0.3	>100	>55.5	Keyaerts et al., 2007
Reserpine	Alkaloids	2.07	15.22	7.3	Wu, 2004
LOA	Agglutinins	2.2 ± 1.3	>100	>45.5	Keyaerts et al., 2007
IRA	Agglutinins	2.2 ± 0.9	50	22.7	Keyaerts et al., 2007
HHA	Agglutinins	3.2 ± 2.8	>100	>31.3	Keyaerts et al., 2007
IRA r	Agglutinins	3.4 ± 2.0	55	16.2	Keyaerts et al., 2007
IRA b	Agglutinins	4.4 ± 3.1	36	8.2	Keyaerts et al., 2007
CA	Agglutinins	4.9 ± 0.8	>100	>20	Keyaerts et al., 2007
NPA	Agglutinins	5.7 ± 4.4	>100	>17.5	Keyaerts et al., 2007
GNA	Agglutinins	6.2 ± 0.6	>100	>16.1	Keyaerts et al., 2007
Escin (Aescin)	Saponins	6.79	16.9	2.5	Wu, 2004
Cladistris	Agglutinins	7.4 ± 0.2	>100	>13.5	Keyaerts et al., 2007
Baicalin	Flavonoids	12.5	>100	>8	Chen et al., 2004
PMRIP m	Agglutinins	18 ± 13	>100	>5.5	Keyaerts et al., 2007
AUA	Agglutinins	18 ± 4	>100	>5.5	Keyaerts et al., 2007
TL M I	Agglutinins	22 ± 6	>50	>2.3	Keyaerts et al., 2007
ML III	Agglutinins	28 ± 11	>100	>12.6	Keyaerts et al., 2007
TL C II	Agglutinins	38 ± 0	>50	>1.3	Keyaerts et al., 2007
LRA	Agglutinins	48	>100	>2.1	Keyaerts et al., 2007
Morniga G II	Agglutinins	50 ± 13	>100	>2	Keyaerts et al., 2007
Glycyrrhizin	Saponins	300 ± 51	>20000	>67	Cinatl et al., 2003

### Natural Components That Inhibit Coronavirus 3CL^pro^ *in vitro*

Coronavirus chymotrypsin-like protease (3CL^pro^) is indispensable for processing viral polyproteins and controlling replicase complex activity (Anand et al., 2003). There are numerous natural components, including triterpenes, flavonoids, polyphenols, glucosinolates, food colorings, and sterols, that are reported to inhibit SARS-CoV 3CL^pro^ (Table 2) (Lin et al., 2005; Ryu et al., 2010a,b; Jo et al., 2020). Eight components are reported to have median inhibitory concentrations (IC_50_) between approximately 1 and 10 μg/ml. Preliminary experimental data show that these compounds have potential for development as anti-2019-nCoV drugs. Some CC_50_ data for these components are available from reports other than those that published the IC_50_ data. Among components with available CC_50_ values, hesperetin had the highest selectivity index (SI) at 328, while sinigrin and aloe-emodin had SI values > 30.

**Table 2:** Potential natural components targeting SARS-nCoV 3CL^pro^.

**Components**	**Categories**	**IC_**50**_ (μg/ml)**	**CC_**50**_ (μg/ml)**	**SI**	**References**
Iguesterin	Triterpenes	1.05 ± 0.12	NM	NM	Ryu et al., 2010b
Hesperetin	Flavonoids	2.5 ± 0.8	820 ± 15	328	Lin et al., 2005
Pristimerin	Triterpenes	2.56 ± 0.31	0.41*	0.16	Ryu et al., 2010b *da Costa et al., 2008
Tingenone	Triterpenes	4.16 ± 0.04	16.83 ± 1.65	4.05	Ryu et al., 2010b Chhetri et al., 2015
Amentoflavone	Flavonoids	4.47 ± 0.65	53 ± 0.9*	11.9	Ryu et al., 2010a *Yin et al., 2014
Celastrol	Triterpenes	4.64 ± 0.09	0.90 ± 0.04*	0.19	Ryu et al., 2010b *Zhang et al., 2018
Luteolin	Flavonoids	5.72 ± 0.63	48.1*	8.41	Ryu et al., 2010a *Dai et al., 2019
Curcumin	Polyphenol	8.66 ± 1.36	11*	1.27	Ryu et al., 2010b *Chen et al., 2013
Herbacetin	Flavonoids	10.03	NM	NM	Jo et al., 2020
Quercetin	Flavonoids	10.67 ± 0.85	199.2*	18.7	Ryu et al., 2010a *Dai et al., 2019
Rhoifolin	Flavonoids	12.31	NM	NM	Jo et al., 2020
Pectolinarin	Flavonoids	23.52	449.0 ± 13.0*	19.1	Jo et al., 2020 *Simões et al., 2011
Dieckol	Phlorotannin	50.6 ± 1.6	>148.5	>2.9	Park et al., 2013
Sinigrin	Glucosinolates	90.1 ± 4.2	>5000	>55.5	Lin et al., 2005
Apigenin	Flavonoids	75.88 ± 5.78	69.2*	0.91	Ryu et al., 2010a *Dai et al., 2019
Aloe emodin	Flavonoids	99.1 ± 2.1	3135 ± 9	31.63	Lin et al., 2005
Indigo	Food colorings	190 ± 2.6	917 ± 18	4.83	Lin et al., 2005
Beta-sitosterol	Sterols	502.1 ± 2.9	613 ± 9	1.22	Lin et al., 2005

### Natural Components Targeting Coronavirus Helicase With Inhibition Activity *in vitro*

SARS-CoV non-structural protein 13 (nsP13) is a helicase that separates dsRNA using the energy of nucleotide hydrolysis (Adedeji et al., 2012) and is a target in screening of antiviral agents. Two natural components of flavonoids, scutellarein and myricetin, are reported to have significant activities, at IC_50_ values <1 μg/ml, in inhibiting SARS-nCoV nsP13, based on screening of eight natural components (Yu et al., 2012) (Table 3). According to other published data, myricetin has an SI value > 116 (Ortega et al., 2017).

**Table 3:** Natural components potentially targeting the SARS-nCoV helicase, nsP13.

**Components**	**Categories**	**IC_**50**_ (μg/ml)**	**CC_**50**_ (μg/ml)**	**SI**	**References**
Scutellarein	Flavonoids	0.25 ± 0.14	NM	NM	Yu et al., 2012
Myricetin	Flavonoids	0.86 ± 0.06	>100*	>116	Yu et al., 2012 *Ortega et al., 2017

### Natural Components With Potential 2019-nCoV Receptor, ACE2, Binding Activity

ACE2 expressed on human cells is the receptor for both SARS-CoV and 2019-nCoV, and considered as a potential target for antiviral drugs (Li et al., 2003; Kuhn et al., 2006; Wrapp et al., 2020). The spike proteins (S-protein) of 2019-nCoV and SARS-CoV share very similar 3-D structures in the receptor-binding domain (RBD), which has a significant ACE2 binding affinity (Lu, 2020; Wrapp et al., 2020; Xu et al., 2020). Molecular docking software has been developed to stimulate the putative binding activity between molecules. Previous results have reported the results for several natural components, including scutellarin, glycyrrhizin, baicalin, flavonoids from citrus fruits, and nicotianamine, with estimated ΔG values ranging from −14.9 to −3.78 kcal/mol (Chen and Du, 2020; Cheng et al., 2020) (Table 4). The residues in ACE2 that contact the S protein RBD of 2019-CoV are 24Q, 30D, 35E, 37E, 38D, 41Y, 42Q, 83Y, 353K, and 393R, which are very similar to that of SARS-Cov (Li et al., 2005a; Lan et al., 2020), and there is no complete coverage of ACE2 binding residues by natural components; however, the residues of ACE2 that bind with glycyrrhizin (559R, 388Q, 393R, and 30D), nobiletin (69W, 351L, and 350D), and neohesperidin (349W, 348A, and 69W) fall partially within the RBD contact region. Therefore, these three natural components may be able to block the binding between 2019-nCoV and its receptor, ACE2.

**Table 4:** Natural components with potential to bind the 2019-nCoV receptor, ACE2.

**Components**	**ΔG (kcal/mol)**	**Sites**	**IC_**50**_ (μg/ml)**	**CC_**50**_ (μg/ml)**	**References**
Scutellarin	−14.9	495E, 957X, 482R	NM	NM	Chen and Du, 2020
Glycyrrhizin	−9.0	559R, 388Q, 393R, 30D	NM	>20000	Chen and Du, 2020
Baicalin	−8.46	149N,273R, 505H	NM	>100	Chen and Du, 2020
Hesperetin	−8.3	613Y, 611S, 482R, 479E	NM	820 ± 15	Chen and Du, 2020
Naringin	−6.85	515Y, 402E, 398E, 394N	NM	2,000	Cheng et al., 2020
Hesperetin	−6.09	562K, 564E, 205G	NM	820 ± 15	Cheng et al., 2020
Naringenin	−6.05	146P, 143L, 131K	NM	NM	Cheng et al., 2020
Nobiletin	−5.42	69W, 351L, 350D	NM	NM	Cheng et al., 2020
Nicotianamine	−5.1	518R, 406E, 409S, 522Q, 442Q	25.5	NM	Chen and Du, 2020
Hesperidin	−4.21	277N, 273R, 505H	NM	NM	Cheng et al., 2020
Neohesperidin	−3.78	349W, 348A, 69W	NM	NM	Cheng et al., 2020

The potential binding of nicotianamine with ACE2 has previously been reported as an ACE2 inhibitor (Takahashi et al., 2015). As the ACE2 catalytic site is distinct from the S-protein-binding domain (Dimitrov, 2003; Li et al., 2003), nicotianamine binding may not block interaction of 2019-nCoV and ACE2; however, it may still act as an inhibitor of 2019-nCoV entry, based on comparisons with N-(2-aminoethyl)-l-aziridine-ethanamine (NAAE) (Adedeji and Sarafianos, 2014), which is an inhibitor of both ACE2 catalytic activity and has antiviral activity, as it inhibits S-protein-induced cell-cell fusion (Huentelman et al., 2004). The antiviral activity of all these natural components requires further investigation. Notably, as mentioned above, glycyrrhizin is reported to inhibit SARS-CoV infection CPE in cell culture (Cinatl et al., 2003). Diammonium glycyrrhizinate (a more absorbable medicinal form of glycyrrhizin) has been approved for clinical trials and recorded with China's National Medical Products Administration (NMPA) for treatment of 2019-nCoV (Yang Y., 2020); its activity may be attributable to ACE2 binding.

### Some Evidence of Natural Components Against 2019-nCoV *in vivo*

Compared with *in vitro* data, *in vivo* experiments are relatively few. So far, only a few studies have reported that natural products can inhibit coronavirus *in vivo*. Initially, Bahrami et al. (2020) demonstrated that Parthenolide could significantly reduce IL (1, 2, 6, and 8) and TNF-α production pathways by using human cell line models, pointing out that Parthenolidemay be one of the herbal candidates of clinical drug for COVID-19. Subsequent, with the help of computer simulation, some new evidences are found. In the study of Zhang et al. (2020), the authors screened the potential anti-virus herbs from the traditional Chinese medicine systems pharmacology (TCMSPT) database (http://www.tcmspw.com/browse.php?qc=herbs). The network pharmacological analysis predicted that at least 26 herbs have potential anti-2019-nCoV effects *in vivo* and can simultaneously regulate host inflammation responses. Similarly, Das et al. (2020) demonstrated that rutin and hesperidin have anti-SARS-CoV-2 ability under *in vivo* condition by using molecular docking approach. In addition, Deng et al. (2020) indicated that PDL (PudilanXiaoyan Oral Liquid, a traditional Chinese medicine preparation composed of Bunge Corydalis, Indigowoad Root, Mongolian Dandelion, and Scutellaria Amoena) exhibited potent anti-SARS-CoV-2 activity *in vivo* by using bioinformatics methods, which may be clinically used for the treatment of pneumonia caused by SARS-CoV-2 infection alone or cocktailed with other effective antivirals. As these studies are based on molecular docking, further *in vivo* validation is needed to study and develop more natural drug against COVID-19.

### Other Functions of Natural Products

In addition to direct resistance to 2019-nCoV infection, medicines of plant origin (Table S1) have numerous other activities, such as antioxidation, eliminating free radicals, anti-inflammatory, and regulation of host immunity and autophagy behavior (Li et al., 2018; Joles, 2020).

Baicalin and scutellarin have wide-spectrum activities anti-RNA viruses, such as MERS and SARS (Chen et al., 2004; Chen and Du, 2020). They against virus effects are strongly associated with supplementary capacity, including anti-oxidative stress, anti-inflammation, and anti-apoptosis potential. Further, *in vitro* experiments have demonstrated that glycyrrhizin can up-regulate nitrous oxide synthase expression, which can help the viral host to eliminate free radicals (Cinatl et al., 2003; Chen and Du, 2020). Meanwhile, given the potential anti-inflammatory activity of flavonoids, citrus fruit and phytochemicals derived from them are promising for prevention and treatment of 2019-nCoV infection (Cheng et al., 2020). Subsequent experiments (including *in vitro* and *in vivo*) shown that another compound, naringin, can inhibit expression of four pro-inflammatory cytokines (COX-2, iNOS, IL-1β, and IL-6) (Cheng et al., 2020). This type of natural product is now listed in the “Diagnosis and Treatment Protocol for Novel Coronavirus Pneumonia” (NHC and SATCM, 2020).

Similar to land plants, some extracts of marine origin also exhibit significant anti-stress and anti-inflammatory abilities. Typical candidates are marine polysaccharides, two of which are griffithsin and fucoidan. Griffithsin, a kind of lectin (secreted by red algae), binds to oligosaccharides on viral glycoproteins surface, including SARS-CoV spike glycoprotein (Zumla et al., 2016). Griffithsin exhibits satisfactory anti-oxidation properties and antitumor activity, which both contribute to its anti-viral efficacy. Fucoidan is a cousin of griffithsin that is widely used to treat liver disease, cancer, and skin infections, due to its anti-inflammatory properties (Dutot et al., 2019). During the SARS outbreak, statistical analyses showed that Shandong Province more actively used fucoidan and recorded a significantly lower mortality rate, relative to other regions, possibly due to its “combined strengthening and elimination” abilities.

Enhancement of immunity is another supporting function of natural products. Clinical studies have demonstrated that natural extracts can greatly improve the immunity of patients and alleviate side effects. Dpo, isolated from *Euphorbia fischerianaSteud*, can stimulate immunity to counteract HSV-1 (Hsu et al., 2016), as well as regulate autophagy, which is also linked to immunity and its anti-HSV-1 effects (Kim et al., 2010). Autophagy is a relatively conserved physiological process, it plays a critical role in maintaining cellular homeostasis. Meanwhile, it also participates in many important physiological processes, including clearance of foreign microorganisms, antigen presentation, and non-specific immune responses (Kim et al., 2010). Autophagy may contribute to resistance to HSV-1 infection by presenting viral antigens on major histocompatibility complex (English et al., 2009).

### Existing Synthetic Drugs and the Relatively Advantages of Natural Products

More attention has been paid to research into, and clinical trials of, synthetic drugs than natural components (Barnard and Kumaki, 2011; Zumla et al., 2016; Lu, 2020); however, due to the rapid development of the pandemic after the 2019-nCoV outbreak, almost no synthetic drugs are available for clinical use against the new disease. We summarize publications detailing *in vitro* tests of typical synthetic drugs after the SARS outbreak (Table 5).

**Table 5:** Potential *in vitro* tests of synthetic drugs for SARS-nCoV.

**Drug name**	**EC_**50**_/IC_**50**_ (μg/ml)**	**CC_**50**_(μg/ml)**	**SI**	**References**
Nelfinavir	0.032 ± 0.016	9.63 ± 1.83	302.1	Yamamoto et al., 2004
Remdesivir	0.042	>6	>140	Sheahan et al., 2017
Chloroquine	1.27 ± 0.17	37.67 ± 2.09	30	Keyaerts et al., 2004
Lopinavir	4	32	8	Chen et al., 2004
Favipiravir*	4.9 ± 2.8	>160	>32	Scharton et al., 2014
Abidol hydrochloride*	8.17 ± 2.14	89.72	11.0	Haviernik et al., 2018
Ribavirin	12.5~200	>1000	5~>80	Cinatl et al., 2003 Chen et al., 2004

*As no data for coronavirus available, the data here for Favipiravir is against Rift Valley fever virus (RVFV) and the data for Abidol hydrochloride is an average of the results for five strains of Zika virus (ZIKV), West Nile virus (WNV), and tick-borne encephalitis virus (TBEV).

Favipiravir, a selective inhibitor of viral RNA-dependent RNA polymerase, was reported as a synthetic drug approved for use in patients with influenza after the 2019-nCoV outbreak and it may be used with care to treat the virus in some circumstances; however, clinic trials are required (Zhang, 2020). Favipiravir has a variable EC_50_ (0.78–4.9 μg/ml) and SI values ranging from >30 to >200 for different RNA viruses (Furuta et al., 2013).

Ribavirin is a guanine derivative approved for treatment of HCV and infection with respiratory syncytial virus (RSV). This compound has a variable EC_50_ and SI when tested against SARS-CoV. As its negative effects on patients with SARS and MERS and side effects, such as anemia, may be serious at high doses, it is doubtful whether it offers sufficient efficacy against 2019-nCoV (Zumla et al., 2016).

The protease inhibitor, lopinavir/ritonavir, is an anti-HIV medicine combination recommended for treatment of early stage disease (Lu, 2020); its EC_50_ is comparable to many mid-level agglutinins of natural components; however, its CC_50_ and SI values are far inferior (Chen et al., 2004). This drug has recently been declared as not recommended for treatment of COVID-19.

Arbidol hydrochloride is a broad spectrum antiviral drug which was recently approved for clinic trials for treatment of 2019-nCoV. It has similar efficacy tolopinavir/ritonavir, but a better SI value (Haviernik et al., 2018).

Chloroquine, an antimalarial drug, is reported to exhibit promising *in vitro* and clinical results against SARS-CoV, and also has an inhibitory impact against 2019-nCoV, with a EC_50_ value of 0.16 μg/ml in Vero E6 cells; it is currently undergoing assessment in an open-label trial (Wang et al., 2020). More than 10 hospitals in different provinces have jointly evaluated the safety and efficacy of chloroquine phosphate. No significant adverse reactions related to the medicine have been detected in more than 100 patients and chloroquine phosphate was reported as effective for treatment of the disease (Song, 2020).

Remdesivir is a novel antiviral drug of the nucleoside analog class. It has a low EC_50_ value against SARS-CoV and MERS-CoV, as well as a high SI value. The drug achieved good efficacy in animal trials and has actually been tested in a medical trial against Ebola. A recent study reported that remdesivir prevented 2019-nCoV (EC_50_ = 0.77 μM in Vero E6 cells) (Wang et al., 2020). Two phase III clinical trials were started in early February 2020 to evaluate intravenous Remdesivir (first day 200 mg and 100 mg/d for 9 days) in patients with 2019-nCoV (Hu and Li, 2020).

The protease inhibitor, nelfinavir, is reported to have a very low EC_50_ value and an SI > 300, which better than that of remdesivir. Nelfinavir is approved and widely used to treat HIV-1. The safety of oral administration for adults of 500 to 750 mg twice per day or 500 to 1,000 mg three times per day for 21 to 28 days is established (Yamamoto et al., 2004); however, the potential of nelfinavir for treatment of 2019-nCoV appears to have been completely ignored.

Compared with synthetic drugs, some natural components have generated superior *in vitro* test data. For example, lycorine may have much better efficacy and safety than any synthetic drugs, including remdesivir and nelfinavir (Wang et al., 2003). Further, APA and myricetin may have much better efficacy and safety than chloroquine, lopinavir, and other synthetic drugs (Xia et al., 2020). Hesperetin and agglutinins with EC_50_values <5 and SI> 30 may have better or equivalent efficacy and safety than synthetic drugs, including chloroquine, lopinavir, favipiravir, arbidol, and ribavirin (Xia et al., 2020). Griffithsin has broad inhibit specturm of CoVs, including SARS-CoV, HCoV-229E, HCoV-OC43, and HCoV-NL63 *in vitro*, as well as in SARS-CoV-infected mice (O'Keefe et al., 2010). In addition, some Himalayan plants (*Justiciaadhatoda, Ocimumbasilicum, Plantago major*, and *Zingiberofficinale*), which contain multiply bioactive substances, such as benzoic, flavonoids, iridoid glycosides, monoterpenoids, sesquiterpenes, triterpenoids, and phenolic compounds, have stronger antiviral activity against adenovirus and influenza virus than chemical drugs (Rahila, 2017). Adams (2020) and Gan (2020) summarized that, relative to chemical drugs, natural products may have broader pharmaco-dynamic mechanisms, including: (i) antiviral effects by inhibiting 2019-nCoV replication or inactivating viral attachment/absorption/penetration abilities; (ii) counteracting 2019-nCoV by regulating cell-autophagy; (iii) exerting anti-viral effects by enhancing host immunity; and (iv) exhibiting significant synergistic effects in combination with synthetic drugs. Regarding the last point, the newest research, coupling traditional Chinese medicine (Qingwen Decoction) and western medicine (Ribavirin) successfully cured 34 patients with 2019-nCoV patients (Xia et al., 2020). These results confirm that combined treatments for 2019-nCoV can significantly reduce the clinical symptoms of patients, shorten the disease course, and improve the clinical cure rate, which warrants promotion and further application (Xia et al., 2020).

### Cases of Use and Precautions Recommended for Natural Products

Since no suitable drug is yet available in the clinic for the treatment of latent 2019-nCoV infection, there is an ongoing search for strategies, based on the prevention of transmission, suppression of reactivation, and viral shedding, together with inhibition of epithelial damage, as effective approaches to progress drug research and development against this virus (Totura and Bavari, 2019). To date, many natural products, including various plants/herbals crude extracts or fractions, have been assessed for their roles against 2019-nCoV. Due to the low toxicity and availability of some active compounds, it is worthwhile to select potential candidates for treatment of 2019-nCoV. To date, application for clinical trials of various natural products are under consideration by the ChiCTR (Chinese Clinical Trail Registry) (Table S2). We have chosen several representative drugs, to discussion their use and precautions.

Baicalin has broad therapeutic efficacy, and there are few reports of it having toxic effects (Ishfaq et al., 2019). Plaque reduction assays showed that baicalin has an EC_50_ of 11 μg/ml in SARS (Chen et al., 2004), while a subsequent study showed that baicalin could inhibit ACE, with an IC_50_ value of 2.24 mM *in vitro* (Deng et al., 2012). Another similar herb is scutellarin, which could reduce the expression and activity of ACE in brain tissue *in vivo* (Wang et al., 2016). Relevant reports indicated no acute cytotoxicity of scutellarin in test cells, and its IC_50_ value against ACE was 48.13 ± 4.98 μM (Wang et al., 2016). These results suggest that baicalin and scutellarin are eco-friendly drugs against SARS viruses. Since 2019-nCoV shares similarity with SARS viruses, we suspect that baicalin and scutellarin are potential candidates for 2019-nCoV treatment. Given the low toxicity of these two natural products, their efficacy against 2019-nCoV warrants further investigation. The standard dose of baicalin for oral administration in humans for SARS, is “~1500 mg (as tablets); or ~6000 mg (calculated from herbs, assuming 30 g of herb used and that the herb contains up to 20% baicalin).” Similarity, the oral protocol for glycyrrhizin is “~300 mg (as tablets) or ~1700 mg (calculated from the herb, assuming that the herb contains 5.65% glycyrrhizin)” (Chen et al., 2004). Compared with the oral method, the recommended intravenous doses for administration of baicalin and glycyrrhizin are approximately 600 and 240 mg, respectively (Chen et al., 2004). For 2019-nCoV, the recommended method for glycyrrhizin administration is a low dose of honeysuckle oral liquid, 60 ml each time, three times a day (ChiCTR2000029954).

Two important herbs that can be sourced from the wild are orange peel (primary active compound, hesperetin) and licorice root (primarily active compound, glycyrrhizin), and these are valuable candidates for treatment of 2019-nCoV. Hesperetin is a bioflavonoid compound abundant in the Chinese medicine, citrus aurantium, which dose-dependently inhibits cleavage activity of the 3CL^pro^ SARS-coronavirus protease in cell-free and cell-based assays, with an IC_50_ of 8.3 μM (Lin et al., 2005). Wu C. R. et al. (2020) using the homology modeling method also confirmed that hesperidin has the potential to inhibit 3CL^pro^ protein and could probably be used for controlling SARS-CoV-2. Similar to hesperetin, glycyrrhizin is another key compound for treatment of respiratory infections. Licorice root (Glycyrrhiza radix) is rich in glycyrrhizin, which is used to treat chronic hepatitis and is relatively non-toxic. It inhibits SARS-CoV adsorption and penetration and was most effective when administered both during and after the viral adsorption period (Cinatl et al., 2003). Given the low toxicity of glycyrrhizin, testing of its efficacy against 2019-nCoV infection is warranted. The recommended method for administration of glycyrrhizinate is an enteric-coated capsules (oral, 150 mg, three times a day), vitamin C tablets (oral, 0.5 g, one a day), alongside standard clinical antiviral treatment (ChiCTR2000029768); however, it should be noted that specific chemical modifications increase the antiviral potency of glycyrrhizin, but also increase its cytotoxicity, thus the SIof the modified form is lower than that of glycyrrhizin (SI ≥ 65) (Hoever et al., 2005).

Plant lectins are natural proteins that target the sugar parts of various glycoproteins. They are widely found in higher plants and are carbohydrate-binding proteins that can specifically recognize and reversible binding to carbohydrates. Initially, lectins were reported to inhibit viral replication by preventing their attachment (Müller et al., 1988); however, subsequent study confirmed that they prevent HIV particles fusion with their target cells (Balzarini et al., 1992). Plant lectins possess marked antiviral properties against both coronaviruses, with EC_50_ values in the lower microgram/ml range (middle nanomolar range), being non-toxic (CC_50_) at 50–100 μg/ml (Keyaerts et al., 2007). For SARS, coronavirus infectivity potential inhibited by lectins specific for the glycans present in the spike glycoprotein, which contains 12 N-glycosylation sites in the SARS-CoV spike protein. The sugars binding to four of these N-glycosylation sites have been confirmed (Krokhin et al., 2003) and the robustest anti-coronavirus activity was appeared among mannose-binding lectins. Besides, a number of glucose-, galactose-, N-acetylgalactosamine-, and N-acetylglucosamine-specific plant agglutinins exhibited anti-coronavirus activity at different degrees. A significant correlation (*r* = 0.7) was found among the EC_50_ values of the mannose-specific plant lectins effective against the two coronaviruses (Keyaerts et al., 2007). Hence, for high-mannose type glycans plants, the recommended daily administration dose is 6–15 g (Pharmacopoeia Commission of PRC, 2015).

Another interesting example is tea, a traditional Chinese drink. The tea extracts, polyphenols (including catechin), have excellent extracellular and intracellular coronavirus inhibition ability *in vitro* (Adem et al., 2020). The first finding was reported in a news from the laboratory in Center for Disease Control of Zhejiang Province (ZJCDC). Their experiments using results showed that 2019-nCoV pre-treated with 2.5–10 mg/mL tea extract had a significant decrease of nucleic acid proliferation rate by 10^4^-10^5^ folds on Vero cell lines. The extracts from green tea at a 0.25 mg/mL (the lowest concentration in their test) could inhibit infection with SARS-CoV-2 on the cell lines. However, due to pressure from public opinion, ZJCDC has withdrawn the news and announced they will arrange more detail investigation (ZJCDC, 2020). Notably, research news issued subsequently from an independent study in Yunnan Agricultural University provided further evidence supporting findings in ZJCDC. Five natural compounds from tea extract were found have affinity for viral S protein, using molecular docking simulation and verified by blocking *in vitro* experiments. The effective monomer molecule, epigallocatechin gallate (EGCG), can bind the 2019-nCoV S protein (Kd = 121 nM) and effectively block the binding of S protein to ACE2 (Yang H., 2020). At present, this research is undergoing clinical trials. This finding provides valuable scientific data for the development of agents for the prevention and treatment of new coronavirus infections. In the 2019-nCoV outbreak in China, we found few cases of infection in Yunnan Province, which may be due to local tea drinking habits (Pu'er tea). Based on the auxiliary functions of tea (detoxification, anti-oxidation, and reduction of the incidence of cardio-cerebrovascular diseases), we believe that the potential of tea compounds against other coronaviruses should not be ignored. Whereas, more clinical research and double-blind randomized trials should be conducted in this area.

In addition to plant extracts, a variety of food materials can be eaten directly, including tangerine peel, fresh citrus fruits, cordate houttuynia, and licorice which are commonly available in daily life, and can be selected as preliminarily materials for emergent self-management programs. Based on the experimental results, Utomo et al. (2020) recommend that *Citrus* sp., followed by galangal, sappan wood, and *Curcuma* sp. can be taken in daily life as prophylaxis of COVID-19. The dosage of ingredients used in lung clearing and detoxifying decoction, published by the state administration of traditional Chinese medicine, or the dosage specified in the Chinese pharmacopeia can be considered as a single or mixed prescription of 5–50 g tangerine peel, is a tisindigoticaroot, or licorice daily. To promote increased immunity, vitamins C and E, small bupleurum, and other specific drugs are recommended as early self-management measures by (Wu and Wen, 2020), and can be supplemented to meet the need for vitamins and other nutrients. Simultaneously, eating more kale, cabbage, broccoli, carrots, and other vegetables containing antiviral active ingredients every day is also highly recommended, as these food are medicinal materials with minimal side effects and great curative potential, easily accessible, and worthy of widespread promotion. Further, it should be emphasized that the possible side effects and safety of natural products should be considered before taking them. Two strategies are key: cleaning the herbal medicine to remove impurities or pollutants and using treatment protocols that account for disease stage and patient condition.

Although the compounds mentioned above exhibit anti-viral activities, additionally evaluation is needed to determine their safe doses in humans by referring to published data from *in vitro* experiments. Since most of the relevant studies only mention the potential anti-2019-nCoV activity of these extracts *in vitro*, future studies need to precisely investigate the mechanisms of antiviral activity of these natural compounds and optimize their utilization. Moreover, it should be noted that, at present, there is no reliable evidence to prove that any one drug is effective against the new coronary pneumonia. Effective clinical decision making requires more than simple observation and empiricism, namely, application of a rigorous set of scientific methods. Scientific practice must be precise, clear, and respectful of objective facts. During drug development, Lindsey Baden, editor of the New England Journal of Medicine, said recently, “one of the challenges is how do we carry out rigorous scientific research when facing a humanitarian crisis disaster? If we follow these scientific rules, it would be a huge step forward”.

## Outlook

The rapid develop of effective interventions anti-2019-nCoV is a big challenge. Based on the existing information on their security and effectiveness against closely related coronaviruses, use of existing antiviral agents among natural products represents a potentially important near-term strategy to tackle 2019-nCoV. In current experiments (some summarized in Table S2), the clinical effect of Chinese herbal medicines currently used in China may be due to these components. Some of these Chinese herbal medicines of which the side effects are clear, the safety has been verified, and the products have already be used in normal diet or health care, such as citrus peel, green tea, liquorice, and Astragalus, etc., could be considered using for early self-intervention approaches against 2019-nCoV, after exposing to a risk of 2019-nCoV, having an asymptomatic infection, or facing limited professional medical resources. To further enhance their therapeutic ability, repurposing these traditional natural drugs and developing new drugs against 2019-nCoV using computer-aided tools are interesting strategies that deserve further consideration in clinical settings. In addition, in the future, we should strengthen several aspects to improve anti-2019-nCoV treatments:

1) Screening of suitable animal models, which are particularly important for testing anti-CoV drugs, as most of these medicines have not been used in humans. Recently, the engineered mice with angiotensin converting enzyme genes has been recommended as a useful model to study COVID-19 (Dediego et al., 2008; Li and Clercq, 2020), relevant animal experiments have also undergoing in some institutions, such as Guangzhou Institutes of Biomedicine and Health (GIBH) (Guangzhou, China).

2) Conduct more clinical trials to identify novel anti-CoV natural product drugs or multidimensional approaches, using methods, such as “herbal medicine + chemical drugs,” “herbal intervention combined with CoV vaccination,” and “the holistic approach.”

3) Prioritization of virus- and host-targeted treatment options for clinical development.

4) Selection of specific natural product formulae, through integrated disease symptom and pathogen-directed approaches, to increase clinical potential.

5) Generate more data on pharmacokinetic and pharmaco-dynamic properties, solubility, metabolic stability, side effects, and dosing regimens. For side effects, some negative effects need attention, such as reserpine and Glycyrrhizin. The former can induce nasal congestion, central nervous system disturb and decline blood pressure (US Food Drug Administration, 2017); the latter can reduction of blood potassium levels and irregular heart rhythm (Curb et al., 1988). Hence, the use of herbal medicines should be guided by viral pathology to a greater extent.

In the long term, the development of new and wide-spectrum antiviral drugs that are active against CoVs probably become the available choice for control circulating and emerging CoV infections. Meanwhile, at present, the Chinese government is promoting treatment with traditional Chinese medicine. Although the difficulties and challenges are fully recognized, we anticipate an increasing contribution and benefits from professionals with expertise in natural drugs, that will provide treatment for patients with pneumonia (Ling, 2020). With the ongoing efforts to prevent the spread of 2019-nCoV worldwide, we believe that a combination of medicinal treatment using natural products and self-intervention can be easily achieved, and could help to prevent social outbreaks of infectious pneumonia.

## Author Contributions

JZ drafted the manuscript. JH and JZ collected and prepared figures and tables. JH completed critical comments and revision. All authors contributed to the article and approved the submitted version.

## Conflict of Interest

The authors declare that the research was conducted in the absence of any commercial or financial relationships that could be construed as a potential conflict of interest.
