# Supplementary material for: Current Findings Regarding Natural Components With Potential Anti-2019-nCoV Activity
Source: Front Cell Dev Biol. 2020 Jul 3;8:589. doi: 10.3389/fcell.2020.00589 (PMC7351523; doi:10.3389/fcell.2020.00589)
Supplement: Supplementary file 1 [file Table_1.DOCX]

Table S1. Plants containing the natural components

| Nature components | Latin name | Common name |
| --- | --- | --- |
| Lycorine, LRA | *Lycoris radiata* | Red spider lily |
| APA | *Allium porrum* | Leek |
| UDA | *Urticadioica* | Stinging nettle |
| Morniga M II, Morniga G II | *Morusnigra* | Black mulberry tree |
| Nictaba | *Nicotiana tabacum* | Tabacco plant |
| EHA | *Epipactis helleborine* | Broad-leaved helleborine |
| Reserpine | *Rauvolfiaserpentine*,*R.vomitoria* | Indian snakeroot |
| LOA | *Listera ovata* | Twayblade |
| IRA, IRA r, IRA b | *Iris hybrid* | Iris |
| HHA | *Hippeastrum hybrid* | Amaryllis |
| CA | *Cymbidium hybrid* | Cymbidium orchid |
| NPA | *Narcissus pseudonarcissus* | daffodil |
| GNA | *Galanthus nivalis* | Snowdrop |
| Escin (Aescin) | *Aesculus hippocastanum* |  |
| Cladistris | *Cladastris lutea* | Yellow wood |
| PMRIP m | *Polygonatummultiflorum monomer* | Solomon’s Seal |
| AUA | *Allium ursinum* | Ramsons |
| TL M I, TL C II | *Tulipa hybrid* | Tulip |
| ML III | *Viscum album* | Mistletoe |
| Glycyrrhizin | *Glycyrrhiza uralensis* |  |
| Iguesterin | *Catha cassinoides* |  |
| Hesperetin, hesperidin, neohesperidin, nobiletin | *Citrus aurantium*,*C.reticulatae* | citrus fruits, citrus peels |
| Pristimerin | *Celastrus aculeatus* |  |
| Tingenone | *Maytenusimbricate* |  |
| Amentoflavone | *Selaginellatamariscina* |  |
| Celastrol | *Tripterygium wilfordii* |  |
| Luteolin | *Reseda odorata* | celery, broccoli, green pepper, parsley, thyme, dandelion, perilla, chamomile tea, carrots, olive oil, peppermint, rosemary, navel oranges, oregano |
| Curcumin | *Zingiberaceae* sp.，*Araceae*sp. |  |
| Herbacetin | *Linumusitatissimum*, *Rhodiola* sp. | common flax, flaxseed, stonecrops |
| Quercetin | *Sabina pingii var. wilsonii* |  |
| Rhoifolin | *Rhodiolacrenulata* |  |
| Pectolinarin | *Cirsium japonicum* |  |
| Dieckol | *Eisenia bicyclis, Ecklonia cava or Ec. stolonifera* |  |
| Sinigrin | *Brassica oleracea*, *B.nigra* | Brussels sprouts, broccoli,black mustard,white mustard, horse radish |
| Apigenin | *Apiumgraveolens* |  |
| Aloeemodin | *Rheum* sp., *Rumex*sp., *Cassia* sp. | rhubarb, patience dock,cassias |
| Indigo | *Polygonum tinctorium* |  |
| Beta-sitosterol |  | Plant, vegetable oil, nuts, avocados, prepared foods |
| Scutellarin, Baicalin | *Scutellariaaltissima*, *S. baicalensis, S. barbara*, *Erigeron breviscapus* |  |
| Myricetin | *Myrica rubra.* |  |
| Naringenin*, Naringin | *Citrussinensis*, *C. maxima*, *C.grandis* | grapefruit, pomelo,bergamot, sour orange, tart cherries, tomatoes, cocoa, Greek oregano, water mint, drynaria, beans |
| Nicotianamine | *Arabidopsis halleri* | Soybean |

Table S2. Applying or ongoing clinical trials of typical natural products drugs

| Drugs | Registration No. | Objectives | Intervention |
| --- | --- | --- | --- |
| 1-Lycorine et al | ChiCTR2000030188 | Evaluation of the efficacy and safety of syndrome differential treatment of COVID-19 according to the severity of the disease | Syndrome differential treatment of Chinese Medicine + Western medicine basic treatment |
| 2- Houttuynia Mixture | ChiCTR2000030168 | To evaluate the positive rate of NCP nucleic acid test on people used Compound Houttuynia Mixture in close contact with novel coronavirus pneumonia(NCP). | Increased Compound Houttuynia Mixture in real world situations |
| 3- Qingwen Baidu Yin | ChiCTR2000030166 | To evaluate the clinical effect of Qingwen Baidu Yin combined with antiviral therapy in the treatment of COVID-19 | (Integrated Chinese and western medicine): Lopinavir-ritonavir tablet combined with recombinant human interferon alpha 2b injection(routine treatment)+Qing-Wen Bai-Du-Yin formula granules |
| 4-soybean extracts | ChiCTR2000030162 | The aqueous extract of soybeans contains a natural product nicotianamine which has been proven to be able to inhibit angiotensin converting enzyme 2 (ACE2) with IC50 of 76nM. ACE2 is crucial to SARS CoV and SARS-CoV-2 fusion to host cell. We hypothesized that aqueous extract of soybeanscould become the major antiviral therapy in SARS-CoV-2 infections. | Daily intake 500ml of aqueous extract from soybeans plus routine treatment |
| 5- dihydroartemisinin piperaquine | ChiCTR2000030082 | The clinical efficacy of dihydroartemisinin piperaquine in the treatment of mild/common novel coronavirus pneumonia (covid-19) was evaluated to provide new effective drugs for the clinical treatment of novel coronavirus and improve the prognosis of this disease. | dihydroartemisinin piperaquine tablets combined with antiviral treatment. |
| 6- Shenfu injection | ChiCTR2000030043 | To evaluate the efficacy and safety of Shenfu injection in the treatment of severe pneumonia caused by novel coronavirus | Conventional treatment and Shenfu injection |
| 7-mixed TCM | ChiCTR2000030034 | 1. Provide effective traditional Chinese medicine treatment protocol for novel coronavirus pneumonia 2. Provide effective TCM preparations against novel coronavirus pneumonia | TCM+Routine treatment of Western Medicine |
| 8-mixed-TCM | ChiCTR2000030027 | In order to find an objective and effective treatment for new type of coronavirus pneumonia (COVID-19), so as to benefit more patients, provide evidence for the clinical efficacy of TCM in the treatment of new type of coronavirus pneumonia. | Western medicine routine treatment plan plus TCM syndrome differentiation treatment |
| 9-Kesuting syru, Keqing capsule | ChiCTR2000029991 | (1) to preliminarily evaluate the clinical efficacy and safety of Kesuting syrup in the in the treatment of mild and moderate novel coronavirus pneumonia (COVID-19); (2) to preliminarily evaluate the clinical efficacy and safety of Keqing capsule in the in the treatment of novel coronavirus pneumonia (COVID-19). | conventional therapy and Keqing capsule, p.o., three pills once, three times a day |
| 10-TCM (Nictaba, luteolin, ect.) | ChiCTR2000029896 | Evaluating the curative effect and prognosis of TCM syndrome differentiation and staging combined with regional characteristics, it can provide clinical reference for the diagnosis and treatment of new coronavirus pneumonia | Prescription based on syndrome differentiation of TCM and Routine treatment of Western Medicine |
| 11-mixed TCM | ChiCTR2000029941 | To Observe the clinical effect of TCM in differentiating and treating novel coronavirus pneumonia | TCM compound granules + western medicine symptomatic treatment |
| 12-mixed-TCM | ChiCTR2000029947 | To observe the clinical effect of Qingyi No. 4 compound on novel coronavirus pneumonia | TCM compound granules + western medicine symptomatic treatment |
| 13- honeysuckle | ChiCTR2000029954 | Evaluate the clinical efficacy of honeysuckle oral liquid in the treatment of NCP; and evaluate the safety of honeysuckle oral liquid in the treatment ofNCP | Basic treatment+Low dose of honeysuckle oral liquid 60ml each time, tid |
| 14-mixed-TCM | ChiCTR2000029956 | Increased resistance during recovery of cured patients of COVID-19, reducing and reducing the risk of repeated infection with COVID-19. | Health Guidance + Guixi Regulating Lung Gong Method + Shenling Baizhu Powder + Moxibustion Treatment |
| 15-vitamin C | ChiCTR2000029957 | To investigate clinical efficacy and safety of high-dose Vitamin C in the treatment of severe and critical severe and critical patients with novel coronavirus pneumonia (COVID-19) to reduce patient mortality. | high-dose Vitamin C＋Standard treatment |
| 16- Xinguan No.1 | ChiCTR2000029637 | To explore the clinical location for Xinguan No.1 in the treatment of 2019-nCoV pneumonia and evaluate its effectiveness and safety. | Xinguan-1 formula＋Standard treatment |
| 17- Glycyrrhizinate | ChiCTR2000029768 | Evaluate the efficacy and safety of Diammonium Glycyrrhizinate Enteric-coated Capsules combined with vitamin C tablets in inpatient with common 2019-nCoV infection in the basic of clinical standard antiviral treatment. | Enteric-coated Capsules (oral, 150mg, Tid), Vitamin C tablets (oral, 0.5g, QD) and clinical standard antiviral treatment |
| 18- shenqi fuzheng | ChiCTR2000029780 | To evaluate the efficacy and safety of shenqi fuzheng injection in the treatment of pneumonia caused by novel coronavirus | Routine treatment and Shenqi Fuzheng Injection |
| 19-mixed TCM | ChiCTR2000029788 | Using a randomized controlled research method, patients with new type of coronavirus pneumonia (COVID-19) were randomly divided into experimental groups and control groups. Interventions were conducted through the cooperative Chinese medicine treatment model, and clinical efficacy evaluation and related experimental indicators were tested. An objective and effective treatment for new coronavirus pneumonia (COVID-19), which will benefit more patients. | Western medicine routine treatment plan plus TCM syndrome differentiation treatment |
| 20-herb medicine | ChiCTR2000029790 | In order to clarify the clinical efficacy of the method of relieving exterior dampness and detoxification in the treatment of dampness toxin depression lung type of novel coronavirus pneumonia, and to provide a scientific basis for improving the Chinese medicine treatment of novel coronavirus pneumonia. | Western medicine basic treatment combined with traditional Chinese medicine |
| 21- thymosin | ChiCTR2000029806 | To explore the efficacy of PD-1 and thymosin in patients with 2019-nCoV severe pneumonia associated with lymphocytopenia | Thymosin for injection 1.6 mg sc qd for 5 days, Camrelizumab 200 mg single dose, diluted to 100 ml intravenous infusion |
| 22- Tanreqing Capsules | ChiCTR2000029813 | To evaluate the efficacy and safety of Tanreqing Capsules in the treatment of pneumonia caused by novel coronavirus | Tanreqing Capsules (oral, 3 capsules at a time, 3 times a day) |
| 23- Xin-Guan-2 | ChiCTR2000029628 | Explore efficacy and safety of Xin-Guan-2 formula in the treatment of suspected 2019-nCoV Caused Pneumonia | Xinguan-2 formula＋Standard treatment |
| 24-herb extracts | ChiCTR2000029439 | To assess the effect of TCM standard decoctions combined with western medicine | TCM standard decoctions+basic western medical therapies |
| 25-TCM | ChiCTR2000029461 | To explore the effect of Therapy of Integrated Traditional Chinese Medicine and Western Medicine for 2019-nCoV Caused Pneumonia (common type) | TCM decoctions+basic conventional therapy |
| 26-TCM | ChiCTR2000029517 | Randomized double-blind placebo-controlled clinical trials were conducted to identify the efficacy and safety of TCM staging against 2019-ncov pneumonia, shortening the recovery time, reducing the clinical symptoms of confirmed cases, and reducing the incidence of critical illness. | placebo |
| 27-plant extract | ChiCTR2000029578 | To identify the efficacy and safety of the Chinese medicine staging program in the cure rate, healing time, reduction of the incidence of severe and critical illness in 2019 new-type coronavirus (2019-nCoV) infection. |  |
